# Prognostic value of glucose to lymphocyte ratio for patients with renal cell carcinoma undergoing laparoscopic nephrectomy: A multi-institutional, propensity score matching cohort study

**DOI:** 10.3389/fsurg.2022.911411

**Published:** 2022-09-29

**Authors:** Jinliang Ni, Ziye Li, Wei Song, Houliang Zhang, Yidi Wang, Yifan Zhang, Haipeng Zhang, Guangcan Yang, Jun Xie, Keyi Wang, Bo Peng, Weipu Mao

**Affiliations:** ^1^ Department of Urology, Shidong Hospital of Yangpu District, Shanghai, China; ^2^ Department of Urology, Shanghai Tenth People’s Hospital, School of Medicine, Tongji University, Shanghai, Shanghai, China; ^3^ Shanghai Clinical College, Anhui Medical University, Shanghai, China; ^4^ Department of Respiratory Medicine, Shanghai Tenth People's Hospital, School of Medicine, Tongji University, Shanghai, China; ^5^ Department of Urology, Affiliated Zhongda Hospital of Southeast University, Nanjing, China

**Keywords:** glucose to lymphocyte ratio, renal cell carcinoma, prognosis, overall survival, cancer-specific survival

## Abstract

**Background:**

We evaluated the prognostic value of preoperative blood glucose to lymphocyte ratio (GLR) in renal cell carcinoma (RCC) patients who underwent laparoscopic nephrectomy through a multi-institutional clinical study.

**Methods:**

A total of 420 patients with RCC from three medical centers from 2014 to 2019 were included in this study. The effect of GLR on overall survival (OS) and cancer-specific survival (CSS) in RCC patients was assessed by Kaplan-Meier survival curves, univariate and multivariate Cox regression analysis. Moreover, a 1:1 propensity score matching (PSM) analysis of different GLR groups was utilized to further confirm the prognostic ability of GLR.

**Results:**

The optimal cut-off value for GLR was 6.8. Patients were divided into high GLR and low GLR groups according to the optimal cut-off value. GLR was significant association with diabetes, cardiovascular disease and AJCC stage. High GLR predicted adverse OS (***P*** = 0.002) and CSS (***P ***< 0.01) in RCC patients. Multivariate Cox regression analysis revealed that high GLR was an independent prognostic factor for OS [hazard ratio (HR): 2.389, 95% confidence interval (CI), 1.136–5.027, ***P*** = 0.008] and CSS (HR: 3.474, 95% CI, 1.555–7.761, ***P*** = 0.002). After PSM analysis of the patients in the high GLR and low GLR groups, high GLR still predicted poor OS (***P*** = 0.021) and CSS (***P ***= 0.037).

**Conclusions:**

High GLR is associated with adverse prognosis in RCC patients, and GLR can serve as an independent prognostic marker for OS and CSS in RCC patients receiving laparoscopic nephrectomy.

## Introduction

As one of the most common malignant tumors of the urinary system, renal cell carcinoma (RCC) accounts for approximately 3% of all new cases of adult malignancies (1). For patients with localized or early stage RCC, surgery with regular follow-up is the predominant modality to treat the tumor, with a 5-year survival rate of approximately 93% (2). It has been reported that more than 30% of patients with RCC were diagnosed when the tumor was locally advanced or metastatic lesions occurred, while 10%–20% of early stage RCC patients also develop recurrence after treatment (3). Because of the presence of metastasis and recurrence, patients suffering from advanced RCC have a poor prognosis, with a 5-year survival rate of only 5%–10% (4). Therefore, establishing an independent prognostic indicator for risk stratification of RCC patients is a clinical challenge that will facilitate tailor optimal treatment strategies and follow-up plans for RCC patients.

Numerous studies have demonstrated that inflammation plays a key role in tumor development and progression (5). Therefore researchers are increasingly interested in the association between inflammatory factors and clinical prognosis of malignant diseases (6). And lymphocytes are one of the markers of systemic inflammatory response and play an important role in cell-mediated inflammation of antitumor response (7). In a variety of malignant diseases, diabetes and elevated blood glucose levels significantly increase the risk of disease in patients, and elevated blood glucose may affect the clinical prognosis of patients (8). Glucose to lymphocyte ratio (GLR), calculated from blood glucose and total lymphocyte count, can reflect human glucose metabolism and systemic inflammatory status, and has been demonstrated to be a good predictor of prognosis for patients with different diseases (9, 10). Nevertheless, studies on the prognostic impact of GLR on RCC patients undergoing laparoscopic nephrectomy are still lacking.

In this study, we aimed to evaluate the predictive value of GLR for overall survival (OS) and cancer-specific survival (CSS) in patients with RCC receiving laparoscopic nephrectomy in a multi-center clinical study.

## Patients and methods

### Patients

In this retrospective study, a total of 590 RCC patients diagnosed with RCC undergoing laparoscopic nephrectomy at Shanghai Tenth People's Hospital, Shanghai Shidong Hospital and Zhongda Hospital Southeast University from January 2014 to December 2019 were collected. The methodology of this study was approved by the all participating institutions Ethics Committee and Institutional Review Board (SHSY- IEC-BG/02.04/04.0-81602469 and ZDKYSB077). And all procedures were complied with the criteria outlined in the Declaration of Helsinki (as revised in 2013). Informed consent was signed by all patients and their relatives who participated in this study.

The selection of all patients included was according to the following criteria: (1) with pathological examination confirmed the diagnosis of RCC; (2) received laparoscopic nephrectomy; (3) complete medical data. The exclusion criteria were as following: (1) age less than 18 years old; (2) underwent other anti-cancer treatments prior to nephrectomy; (3) with other serious malignancies affecting survival or diseases causing increased blood glucose levels; (4) missing follow-up data or clinicopathological data. After exclusion by the above criteria, a total of 170 patients were excluded, leaving 420 patients included in this study.

### Clinicopathological variables and follow-up

Patient related clinicopathological data were accessed and obtained from the electronic medical records of relevant institutions. Clinical variables including hypertension, age, smoking, gender, diabetes, BMI and cardiovascular diseases were collected in this study. Pertinent blood samples were obtained preoperatively and relevant laboratory data were gathered. Assessment of laboratory test data 2 days prior to surgery or closest to the time of surgery. The AJCC stage, TNM stage, Fuhrman classification of the patients were diagnosed by the pathology report and relevant data were collected. The blood glucose-to-lymphocyte ratio (GLR) was defined as the ratio of preoperative fasting glucose (mmol/L) to lymphocyte count (×10^9^/L). Clinic records or telephone follow-up were performed for the patients included in this study. After treatment, follow-up was performed every 3 months for the first 2 years and every 6 months thereafter. The time of the last follow-up visit or the time of death was defined as the patient follow-up endpoint, and the date and reason were recorded. The time from the date of surgical treatment to the date of death or the last follow-up visit was calculated as overall survival (OS). Cancer-specific survival (CSS) was calculated from the time between the date of curative resection and the date of death due to RCC.

### Statistical analysis

SPSS software (version 24.0), RStudio software (version 1.2.5033) and Graphpad Prism (version 8.3.0) were used for the all statistical data analysis of this study. Statistical results with ***P*** < 0.05 were considered statistically significant.

X-tile is a new bioinformatics tool for biomarker evaluation and result-based cut-point optimization (11). The optimal cut-off level for GLR was determined by using X-tile program (version 3.6.1). We divided the GLR patients into high and low groups based on optimal cut-off level. The categorical variables for baseline characteristics in this study were expressed as frequency (percentage). Categorical variables for baseline characteristics between different groups were analyzed using the *chi-square* test or Fisher's exact test, and continuous variables were analyzed using the Student's *t*-test. Correlation between GLR and clinicopathological characteristics was assessed by performing *Chi-square* test or Kruskal-Wallis test. Cox proportional hazards regression models were performed for univariate and multivariate analyses to identify independent prognostic factors for OS and CSS. The relevant unadjusted hazard ratios (HR) and 95% confidence intervals (CI) were calculated from Cox regression models to assess the association of GLR with OS and CSS. In multivariate Cox regression analysis, two models were constructed to further evaluate the relationship between GLR with OS and CSS. Basic model: age, gender, BMI, hypertension, diabetes, cardiovascular diseases, and smoking; extended model: basic model plus AJCC stage, T stage, N stage, M stage, and Fuhrman grade. For further validation of the model, we randomly divided the total cohort into a training cohort (*n* = 289) and a validation cohort (*n* = 124) in the ratio of 7:3. OS and CSS of patients in different GLR groups were compared by Kaplan-Meier method, and the correlation of GLR with OS and CSS was assessed by log-rank test. We performed PSM analysis for the included patients to balance the differences in some variables between the two groups. A 1:1 probabilistic score matching (PSM) analysis was performed for the high GLR and low GLR groups using the R software “Matching” package. Patients were adjusted for age, gender, BMI, hypertension, diabetes, cardiovascular diseases, smoking, AJCC stage, T-stage, N-stage, M-stage and Fuhrman grade. Further analysis of the effect of GLR on OS and CSS in RCC patients.

## Results

As illustrated in Supplementary Figure S1, the optimal cut-off value for GLR was 6.8. The cut-off point of GLR for the maximum *χ*^2^ log-rank value was 10.928 (***P*** = 0.025). This study included 420 RCC patients received laparoscopic nephrectomy who were divided into a high GLR group (*n* = 376, 89.5%) and a low GLR group (*n* = 44, 10.5%). A summary of the baseline clinical and pathological characteristics of patients in different GLR subgroups was presented in Table 1. The results showed that diabetes, cardiovascular disease and AJCC IV stage were more frequently observed in the high GLR group relative to the low GLR group (all ***P ***< 0.05). In addition, patients in the high GLR group had a higher incidence of perioperative complications compared to the low GLR group.

**Table 1:** Baseline characteristics of the patients according to GLR.

Characteristic	GLR		*P*-value
	Low group	High group	
Total	376 (89.5)	44 (10.5)	
Age, y			0.330
≤65	274 (72.9)	29 (65.9)	
>65	102 (27.1)	15 (34.1)	
Gender			0.768
Male	248 (66.0)	30 (68.2)	
Female	128 (34.0)	14 (31.8)	
BMI categorized, kg/m^2^			0.936
<25	216 (57.4)	25 (56.8)	
≥25	160 (42.6)	19 (43.2)	
Hypertension			0.688
No	217 (57.7)	24 (54.5)	
Yes	159 (42.3)	20 (45.5)	
Diabetes			<0.001
No	326 (86.7)	28 (63.6)	
Yes	50 (13.3)	16 (36.4)	
Cardiovascular diseases			0.047
No	337 (89.6)	35 (79.5)	
Yes	39 (10.4)	9 (20.5)	
Smoking			0.705
No	316 (84.0)	36 (81.8)	
Yes	60 (16.0)	8 (18.2)	
AJCC stage			0.032
I	282 (75.0)	29 (65.9)	
II	23 (6.1)	2 (4.5)	
III	53 (14.1)	6 (13.6)	
IV	18 (4.8)	7 (15.9)	
T-stage			0.263
T1	287 (76.3)	30 (68.2)	
T2	26 (6.9)	3 (6.8)	
T3	55 (14.6)	8 (18.2)	
T4	8 (2.1)	3 (6.8)	
N-stage			0.053
N0	364 (96.8)	40 (90.9)	
N1	12 (3.2)	4 (9.1)	
M-stage			0.096
M0	362 (96.3)	40 (90.9)	
M1	14 (3.7)	4 (9.1)	
Fuhrman grade			0.118
I	65 (17.3)	5 (11.4)	
II	231 (61.4)	30 (68.2)	
III	73 (19.4)	6 (13.6)	
IV	7 (1.9)	3 (6.8)	
Complications			
Hematuria	12 (3.2)	1 (2.3)	
Infection	20 (5.3)	2 (4.5)	

GLR, glucose-to-lymphocyte ratio; BMI, Body mass index; AJCC, American Joint Committee on Cancer.

**Figure 1:**
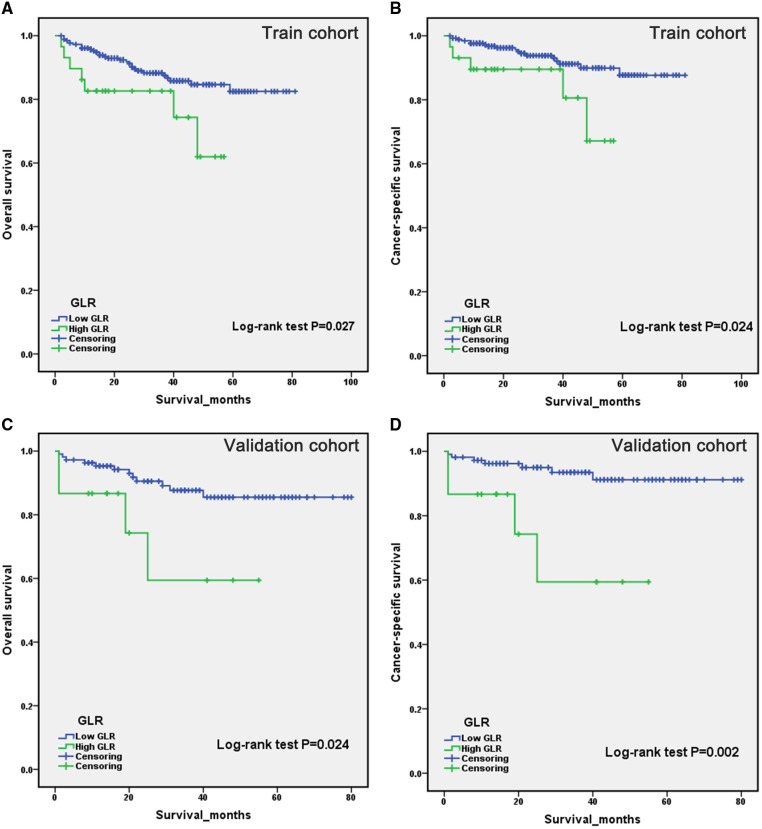
Kaplan-Meier curves for overall survival and cancer-specific survival of patients with renal cell carcinoma after laparoscopic nephrectomy in the train cohort (**A,B**) and validation cohort (**C,D**), stratified by glucose to lymphocyte ratio.

Patients in the high GLR and low GLR groups were compared by Kaplan-Meier analysis. Survival analysis revealed that patients with low GLR had a longer survival time than those with high GLR. Kaplan-Meier curve indicated that patients in the high GLR group were associated with worse OS (***P*** = 0.027 and ***P ***= 0.024, respectively) and CSS (***P*** = 0.024 and ***P ***= 0.002, respectively) compared to the low GLR group in both the training and validation cohorts (Figure 1).

Subsequently, we analyzed the association among different clinical characteristics with OS and CSS by univariate and multivariate Cox regression. Univariate Cox regression analysis showed that high BMI level, AJCC III and IV stages, T3 and T4 stage, N1 stage, M1 stage, Fuhrman III and IV grades, and high GLR (all ***P*** < 0.05) were associated with worse OS when BMI < 25 kg/m^2^, AJCC I stage, T1 stage, N0 stage, M0 stage, Fuhrman I grade and low GLR were taken as reference, respectively (Table 2). Multivariate Cox regression analysis revealed that GLR was consistently an independent risk factor for OS, in both the basic model (low GLR vs. high GLR: HR = 2.784, 95% CI, 1.429–5.423, ***P*** = 0.003) and extended model (low GLR vs. high GLR: HR = 2.621, 95% CI, 1.321–5.201, ***P*** = 0.006). As well, factors related to CSS were analyzed. As shown in Table 3, taking AJCC I stage, T1 stage, N0 stage, M0 stage, Fuhrman I grade and low GLR as a reference, univariate Cox regression analysis demonstrated that AJCC III and IV stages, T2, 3 and T4 stage, N1 stage, M1 stage, Fuhrman III and IV grades, and high GLR were associated with worse CSS (all ***P*** < 0.05). GLR was always an independent risk factor for CSS, similarly in the basic model (low GLR vs. high GLR: HR = 3.717, 95% CI, 1.724–8.014, ***P*** = 0.001) and extended model (low GLR vs. high GLR: HR = 4.080, 95% CI, 1.842–9.040, ***P*** = 0.001).

**Table 2:** Univariate and multivariate analyses of factors associated with overall survival (OS).

Characteristics	Univariate analysis		Basic model		Extended model	
	Hazard Ratio (95% CI)	*P*-value	Hazard Ratio (95% CI)	*P*-value	Hazard Ratio (95% CI)	*P*-value
Age, y						
≤65	Reference		Reference		Reference	
>65	1.645 (0.937–2.886)	0.083	–	0.102	–	0.489
Gender						
Male	Reference		Reference		Reference	
Female	1.162 (0.658–2.050)	0.605	–	0.527	2.060 (1.127–3.763)	0.019
BMI categorized, kg/m^2^						
<25	Reference		Reference		Reference	
≥25	0.507 (0.274–0.938)	0.030	–	0.066	–	0.279
Hypertension						
No	Reference		Reference		Reference	
Yes	1.030 (0.589–1.801)	0.917	–	0.691	–	0.453
Diabetes						
No	Reference		Reference		Reference	
Yes	0.573 (0.228–1.444)	0.238	–	0.138	–	0.068
Cardiovascular diseases						
No	Reference		Reference		Reference	
Yes	1.231 (0.554–2.733)	0.610	–	0.636	–	0.635
Smoking						
No	Reference		Reference		Reference	
Yes	1.267 (0.635–2.529)	0.503	–	0.541	–	0.890
AJCC stage						
I	Reference				Reference	
II	1.178 (0.274–5.060)	0.826			–	0.957
III	4.524 (2.298–8.906)	<0.001			–	<0.001
IV	11.613 (5.898–22.864)	<0.001			–	0.116
T-stage						
T1	Reference				Reference	
T2	1.935 (0.663–5.644)	0.227			–	0.342
T3	5.987 (3.288–10.900)	<0.001			–	0.667
T4	5.136 (1.760–14.987)	0.003			–	0.913
N-stage						
N0	Reference				Reference	
N1	4.611 (2.076–10.242)	<0.001			–	0.202
M-stage						
M0	Reference				Reference	
M1	12.168 (6.542–22.632)	<0.001			7.503 (2.151–26.167)	0.002
Fuhrman grade						
I	Reference				Reference	
II	1.686 (0.648–4.384)	0.284			–	0.531
III	3.079 (1.106–8.568)	0.031			–	0.874
IV	16.337 (4.620–57.769)	<0.001			–	0.014
GLR						
Low group	Reference		Reference		Reference	
High group	2.784 (1.429–5.423)	0.003	2.784 (1.429–5.423)	0.003	2.621 (1.321–5.201)	0.006

OS, Overall survival; CI, confidence interval; BMI, Body mass index; AJCC, American Joint Committee on Cancer; GLR, glucose-to-lymphocyte ratio.

Adjusted covariates: Basic model: age, gender, BMI, hypertension, diabetes, cardiovascular diseases, and smoking; Extended model: core model plus AJCC stage, T stage, N stage, M stage, and Fuhrman grade.

**Table 3:** Univariate and multivariate analyses of factors associated with cancer-specific survival (CSS).

Characteristics	Univariate analysis		Basic model		Extended model	
	Hazard Ratio (95% CI)	*P*-value	Hazard Ratio (95% CI)	*P*-value	Hazard Ratio (95% CI)	*P*-value
Age, y						
≤65	Reference		Reference		Reference	
>65	1.343 (0.647–2.786)	0.428	–	0.666	–	0.488
Gender						
Male	Reference		Reference		Reference	
Female	0.884 (0.418–1.867)	0.746	–	0.977	–	0.091
BMI categorized, kg/m^2^						
<25	Reference		Reference		Reference	
≥25	0.531 (0.245–1.148)	0.107	–	0.098	–	0.243
Hypertension						
No	Reference		Reference		Reference	
Yes	1.920 (0.953–3.868)	0.068	2.099 (1.058–4.166)	0.034	3.162 (1.536–6.508)	0.002
Diabetes						
No	Reference		Reference		Reference	
Yes	0.755 (0.265–2.155)	0.600	–	0.211	–	0.715
Cardiovascular diseases						
No	Reference		Reference		Reference	
Yes	0.808 (0.246–2.655)	0.726	–	0.556	–	0.744
Smoking						
No	Reference		Reference		Reference	
Yes	1.447 (0.626–3.345)	0.388	–	0.223	–	0.871
AJCC stage						
I	Reference				Reference	
II	2.717 (0.576–12.813)	0.207			–	0.541
III	6.532 (2.518–16.942)	<0.001			–	0.172
IV	23.676 (9.806–57.167)	<0.001			–	0.460
T-stage						
T1	Reference				Reference	
T2	4.375 (1.345–14.238)	0.014			–	0.125
T3	9.677 (4.226–22.157)	<0.001			–	0.686
T4	11.714 (3.596–38.162)	<0.001			–	0.143
N-stage						
N0	Reference				Reference	
N1	5.396 (2.076–14.027)	0.001			3.169 (1.169–8.587)	0.023
M-stage						
M0	Reference				Reference	
M1	19.428 (9.398–40.162)	<0.001			13.693 (6.295–29.788)	<0.001
Fuhrman grade						
I	Reference				Reference	
II	2.426 (0.554–10.635)	0.240			2.043 (0.461–9.056)	0.347
III	6.274 (1.386–28.395)	0.017			4.438 (0.949–20.753)	0.058
IV	15.715 (3.298–42.524)	<0.001			14.352 (2.401–85.810)	0.004
GLR						
Low group	Reference		Reference		Reference	
High group	3.850 (1.787–8.298)	0.001	3.717 (1.724–8.014)	0.001	4.080 (1.842–9.040)	0.001

CSS, Cancer-specific survival; CI, confidence interval; BMI, Body mass index; AJCC, American Joint Committee on Cancer; GLR, glucose-to-lymphocyte ratio.

Adjusted covariates: Basic model: age, gender, BMI, hypertension, diabetes, cardiovascular diseases, and smoking; Extended model: core model plus AJCC stage, T stage, N stage, M stage, and Fuhrman grade.

**Figure 2:**
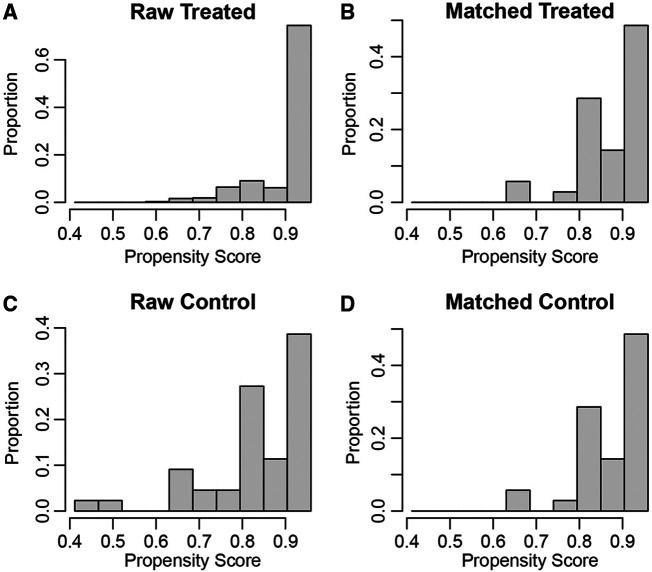
The propensity score results for overall survival (**A,B**) and cancer-specific survival (**C,D**) after propensity score matching.

**Figure 3:**
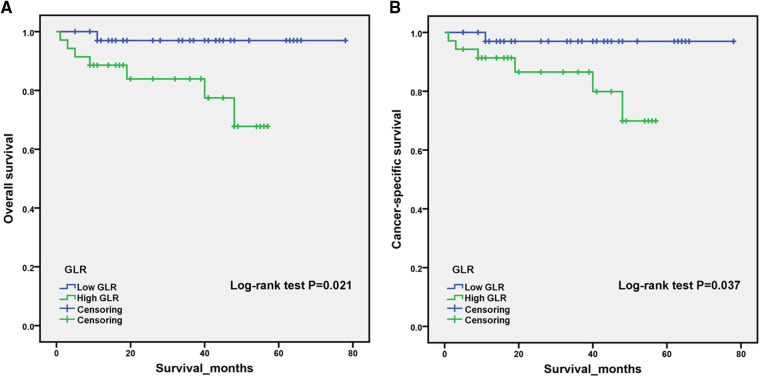
After propensity score matching, Kaplan–Meier curves for overall survival (**A**) and cancer-specific survival (**B**) in patients stratified according to glucose to lymphocyte ratio.

PSM analysis is a statistical method commonly used to reduce the effect of confounding factors by implementing the removal of confounding bias from the observation cohort when randomization is not possible (12, 13). To exclude the effect of other confounding variables, we performed a 1:1 PSM analysis of patients in different GLR subgroups. We adjusted for the 12 variables of age, gender, BMI, hypertension, diabetes, cardiovascular diseases, smoking, AJCC stage, T-stage, N-stage, M-stage and Fuhrman grade (Figure 2). After PSM analysis, the high GLR group and low GLR group included 35 patients, respectively. We subsequently performed a survival analysis of 70 patients and the Kaplan-Meier curve demonstrated consistent results as before. Patients in the high GLR group had worse OS (***P*** = 0.021) and CSS (***P*** = 0.037) than those in the low GLR group (Figure 3).

## Discussion

In the present study, we evaluated for the first time the prognostic value of GLR, in patients with RCC receiving laparoscopic nephrectomy. A total of 420 patients were included in this multi-institutional retrospective study, and we investigated the association between patients with different subgroups of GLR and OS and CSS by Kaplan-Meier curves, univariate and multivariate Cox regression analyses. The results indicated that high GLR was correlated with worse prognosis compared to low GLR and GLR was an independent prognostic factor for OS and CSS. Further 1:1 PSM analysis of the patients was subsequently performed and the results remained consistent. Patients with elevated GLR were associated with poorer OS and CSS.

RCC is the most common type of kidney cancer, comprising approximately 3%–5% of adult malignancies, with a poor prognosis and a 5-year relative survival rate of 75.2% (14). An increasing number of studies have demonstrated that the tumor microenvironment plays an essential role in the development of malignant tumors (15). Lymphocytes are an important component of the tumor microenvironment and inhibit tumor proliferation, invasion, and metastasis by mediating anti-tumor immune responses, inducing apoptosis, and producing cytotoxicity against tumor cells (16). Previous studies have analyzed preoperative blood counts from 430 ccRCC patients treated with primary surgery. The results indicated that lymphopenia was associated with poorer OS, independent of tumor grade, stage, age, smoking and comorbidity index (17). In RCC, various inflammatory indicators consisting of multiple inflammatory cells, such as SIRI and LMR, have been demonstrated to be independent predictors of prognosis (18, 19).

Blood glucose as a metabolic marker is involved in the development of chronic subclinical inflammation (20). Several studies have demonstrated that abnormal glucose regulation promotes tumor proliferation, invasion and migration, inhibits tumor cell apoptosis and increases resistance to chemotherapeutic agents (21). Hyperglycemia can cause hyperinsulinemia, increase the level of reactive oxygen species (ROS), and promote the pathway of epithelial-to-mesenchymal transition (EMT), thereby promoting the progression of malignancy (22). Poor glycemic control in patients with hepatitis C virus (HCV)-associated hepatocellular carcinoma (HCC) with diabetes mellitus after curative resection was strongly associated with postoperative tumor recurrence (23). The results of a single-center retrospective study demonstrated that diabetes was a predictor of recurrence after surgery for non-metastatic renal cell carcinoma, especially in obese patients (24). He et al. found that higher preoperative blood glucose levels were associated with prolonged patient length of stay by collecting clinical data from 338 RCC patients undergoing laparoscopic nephrectomy (25). In patients with metastatic renal cell carcinoma (mRCC), CSS and OS were significantly shorter in preoperative diabetic patients than in non-diabetic patients. Not only that, diabetic patients with good glycemic control had better oncologic outcomes compared to those with poor glycemic control (26).

GLR combined with preoperative glucose and lymphocytes served as a comprehensive biomarker to predict the disease prognosis. Chen et al. (27) demonstrated that GLR was a reliable prognostic factor and that higher GLR significantly enhanced the in-hospital mortality risk in critically ill patients with acute pancreatitis. In addition, pre-treatment GLR might predict OS in patients with inoperable pancreatic cancer (28). Zhang et al. (29) analyzed clinical data from 259 patients with pancreatic ductal adenocarcinoma and found that GLR was a prognostic predictor for patients with PDAC who underwent radical surgery. However, to our knowledge, the present study was the first to investigate the association of GLR with OS and CSS in patients with RCC undergoing laparoscopic nephrectomy and GLR could be used as an independent prognostic factor.

As a retrospective study, the results of this study were positive, but with unavoidable limitations. First of all, although this study was based on a multicenter study conducted at three institutions, a larger sample size is needed for prospective studies for validation. Second, data on the preoperative nutritional status of the patients were lacking. Finally, other treatments were not included in the study.

## Conclusions

Overall, we demonstrated for the first time that GLR is associated with poor prognosis in patients with RCC receiving laparoscopic nephrectomy. GLR can serve as an independent prognostic factor for OS and CSS in patients with RCC, providing clinicians with a useful indicator to plan treatment strategies.

## Data Availability

The original contributions presented in the study are included in the article/**Supplementary Material**, further inquiries can be directed to the corresponding author/s.
