# Supplementary figures and images for: Prognostic value of glucose to lymphocyte ratio for patients with renal cell carcinoma undergoing laparoscopic nephrectomy: A multi-institutional, propensity score matching cohort study
Source: Front Surg. 2022 Sep 29;9:911411. doi: 10.3389/fsurg.2022.911411 (PMC9556963; doi:10.3389/fsurg.2022.911411)

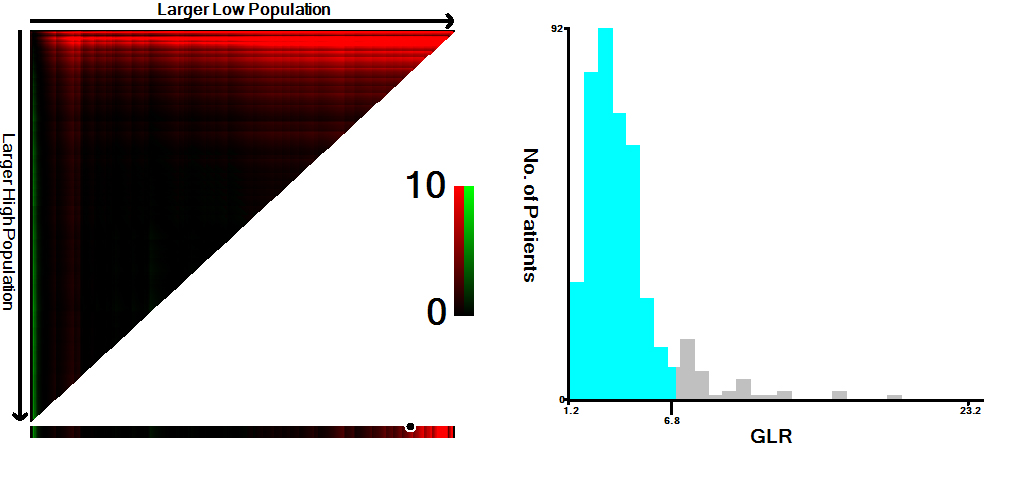

Supplement: Supplementary file 1 [file Image1.jpeg]
